# A semisynthetic borrelidin analogue BN-3b exerts potent antifungal activity against *Candida albicans* through ROS-mediated oxidative damage

**DOI:** 10.1038/s41598-020-61681-0

**Published:** 2020-03-19

**Authors:** Hao Su, Caijuan Hu, Bixuan Cao, Xiaodan Qu, Peipei Guan, Yu Mu, Li Han, Xueshi Huang

**Affiliations:** Institute of Microbial Pharmaceuticals, College of Life and Health Sciences, Northeastern University, Shenyang, 110819 P.R. China

**Keywords:** Mechanism of action, Target identification

## Abstract

In the process of investigating the antifungal structure-activity relationships (SAR) of borrelidin and discovering antifungal leads, a semisynthetic borrelidin analogue, BN-3b with antifungal activity against *Candida albicans*, was achieved. In this study, we found that oxidative damage induced by endogenous reactive oxygen species (ROS) plays an important role in the antifungal activity of BN-3b. Further investigation indicated that BN-3b stimulated ROS accumulation, increased malondialdehyde (MDA) levels, and decreased reduced/oxidized glutathione (GSH/GSSG) ratio. Moreover, BN-3b decreased mitochondrial membrane potential (MMP) and ATP generation. Ultrastructure analysis revealed that BN-3b severely damaged the cell membrane of *C. albicans*. Quantitative PCR (RT-qPCR) analysis revealed that virulence factors of *C. albicans SAP*s, *PLB1*, *PLB2*, *HWP1*, *ALS*s, and *LIP*s were all down-regulated after BN-3b exposure. We also found that BN-3b markedly inhibited the hyphal formation of *C. albicans*. In addition, *in vivo* studies revealed that BN-3b significantly prolonged survival and decreased fungal burden in mouse model of disseminated candidiasis.

## Introduction

*Candida albicans* is an opportunistic fungal pathogen^1^, causing skin and mucosal infections in healthy individuals^2^. Moreover, *C. albicans* can cause fatal systemic disease when immune function is damaged^3^. Disseminated candidiasis caused by *C. albicans* is the main cause of death in immunocompromised patients^3,4^.

Borrelidin (BN), an 18-membered macrolide polyketide^5^, was isolated from the fermentation broth of *Streptomyces vinaceusdrappus*^6^. In the process of investigating the antifungal SAR of BN and discovering antifungal leads, forty-seven borrelidin derivatives (BNs) were synthesized by our research group^5^. Among them, a BN ester analogue BN-3b was greatly promising antifungal candidate. The MIC (minimum inhibitory concentration) values of BN-3b against *C. albicans* and *Candida parapsilosis* were 50 μg/mL and 12.5 μg/mL, respectively (Table 1). In this study, we will explore the antifungal mechanism of BN-3b.

**Table 1:** *In vitro* antifungal activity of BN-3b.

Strains	BN-3b		FLC	AMB	
	MIC^a^ (μg/mL)	MIC_80_^b^ (μg/mL)	MIC_80_^b^ (μg/mL)	MIC^a^ (μg/mL)	MIC_80_^b^ (μg/mL)
*C. albicans* SC5314	50	12.5	2.0	2.0	0.5
*C. albicans* CGMCC 2.2086	50	12.5	2.0	2.0	0.5
*C. parapsilosis*	12.5	3.13	2.0	1.0	0.5
*C. neoformans*	>100.0	>100.0	4.0	2.0	0.25
*A. niger*	>100.0	>100.0	>8.0	2.0	0.5
*R. solani*	>100.0	>100.0	>8.0	8.0	2.0
*F. oxysporum*	50	12.5	>8.0	2.0	0.5
*A. alternata*	50	12.5	>8.0	2.0	0.5
*A. fumigatus*	50	12.5	>8.0	2.0	0.5
*B. cinerea*	25	6.25	>8.0	2.0	0.25

^a^MIC was defined as the minimal inhibitory concentration of a compound. Identical values were obtained for each compound in three replicates by visual investigation as stated in the experimental section.

^b^The drug MIC_80_ was defined as the first well with an approximate 80% reduction in growth compared to the growth of the drug-free well.

^c^Fluconazole (FLC) and ^d^amphotericin B (AMB) were served as the positive controls.

Several studies have suggested that endogenous ROS mediated oxidative damage participate in the antifungal activity of amphotericin B (AMB) and fluconazole (FLC)^7–11^. These findings implied that oxidative stress involved in the antifungal mechanism of antifungal agents. ROS are the byproducts of cellular metabolism and mainly produced in the mitochondria^12^. However, overproduction of ROS resulted in damage of nucleic acids, proteins, and lipids^4^.

*C. albicans* has developed an effective battery of virulence factors^13^ that promote disease establishment and progression^14^. Among these virulence factors, lipases (LIPs), phospholipases, agglutinin-like sequences (ALSs), secreted aspartyl proteinases (SAPs), and hyphal wall protein (HWP1) are most significant in virulence^14–17^. Fungal virulence factors are potential targets for drug development^15^. In this study, we determined the expression of virulence factors (*SAP*s, *PLB1*, *PLB2*, *HWP1*, *ALS*s, and *LIPs*) of *C. albicans* after exposure to BN-3b using RT-qPCR.

The mouse model of disseminated candidiasis has been used extensively to study antifungal drug efficacy^18^. In current study, we also evaluated the efficacy of BN-3b in mouse model of disseminated candidiasis caused by *C. albicans*.

## Results

### *In vitro* antifungal activities of BN-3b

**Figure 1:**
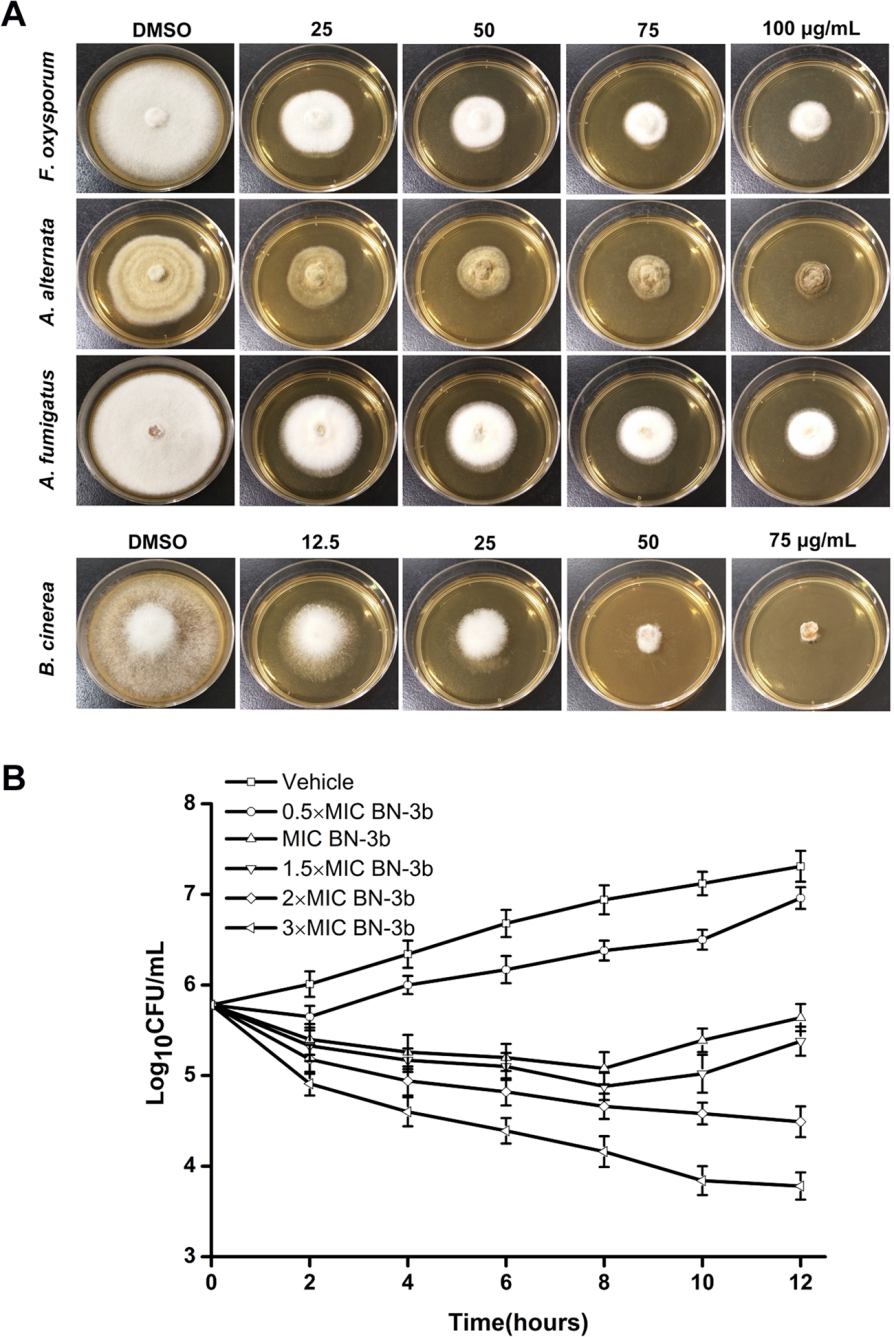
Antifungal activities of BN-3b. (**A**) BN-3b effected on mycelial growth of fungus. Photographs indicating petri dishes containing each fungal species including *F. oxysporum*, *A. alternata*, *A. fumigatus*, and *B. cinerea* were taken by digital camera at 4 days after inoculation. (**B**) *C. albicans* SC5314 growth in time-dependent kill curve assay. *C. albicans* SC5314 cells were treated with BN-3b (0.5 × MIC, MIC, 1.5 × MIC, 2 × MIC and 3 × MIC, respectively) for 12 h. The Log_10_CFU/mL is plotted versus time. Data represent the mean ± SD of three independent experiments. BN-3b showed significant inhibition of proliferation for *C. albicans* cells in a dose-dependent manner (*p* < 0.01).

The antifungal activities of BN-3b were evaluated according to the Clinical and Laboratory Standards Institute (CLSI) guidelines. BN-3b showed strong antifungal activity against *C. albicans* SC5314, *C. albicans* CGMCC 2.2086 and *C. parapsilosis*, with MIC values of 50, 50, and 12.5 μg/mL, respectively (Table 1). In addition, BN-3b displayed antifungal activity against a wide range of plant pathogenic fungi and inhibited mycelial growth of fungus including *Fusarium oxysporum*, *Alternaria alternata*, *Aspergillus fumigatus*, and *Botrytis cinerea* in a dose-dependent manner (Fig. 1A). The MIC values of BN-3b against *F. oxysporum*, *A. alternata*, *A. fumigatus*, and *B. cinerea* were 50, 50, 50, and 25 μg/mL, respectively (Table 1). In order to further evaluate the effect of BN-3b on *C. albicans*, an analysis of fungal viability was carried out by CFU counting (time kill curves; Fig. 1B). The results indicated treatment with BN-3b at 0.5 × MIC reduced the cell viability of *C. albicans* SC5314 even after 2 h of treatment compared with the vehicle group; treatment with BN-3b at MIC and 1.5 × MIC exhibited fungicidal activity against *C. albicans* SC5314 in a dose and time-dependent manner within 8 h; BN-3b at higher concentrations (2 × MIC and 3 × MIC) decreased yeast viability in a time-dependent manner even after 8 h (Fig. 1B).

### Effect of BN-3b on ultrastructure of *C. albicans*

**Figure 2:**
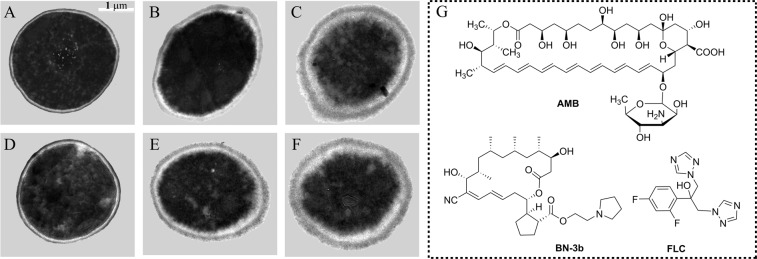
Ultrastructure of *C. albicans* SC5314 cell. *C. albicans* SC5314 were treated with BN-3b or vehicle and were observed by transmission electron microscopy. (**A**) vehicle treatment; (**B**) treated with 25.0 μg/mL of FLC; (**C**) treated with 2.0 μg/mL of AMB; (**D**) treated with 25.0 μg/mL of BN-3b; (**E**) treated with 50.0 μg/mL of BN-3b; (**F**) treated with 75.0 μg/mL of BN-3b; (**G**) structures of BN-3b, FLC, and AMB. The cell membrane of *C. albicans* was seriously destroyed by BN-3b, FLC or AMB. The white bar represents a length of 1 μm.

We next utilized transmission electron microscopy to reveal ultrastructure of *C. albicans* SC5314 cells after treated with BN-3b. As shown in Fig. 2, BN-3b treated cells exhibited obvious alteration in the morphology compared to vehicle. *C. albicans* SC5314 cells showed normal cellular morphology with a distinct cell wall and an intact cell membrane in vehicle treated group (Fig. 2A). In contrast, the cell membrane of *C. albicans* was seriously destroyed after treated with BN-3b (Fig. 2D–F). The results indicated that BN-3b killed fungi through destroying the cell membrane.

### BN-3b inhibited *C. albicans in vivo*

**Figure 3:**
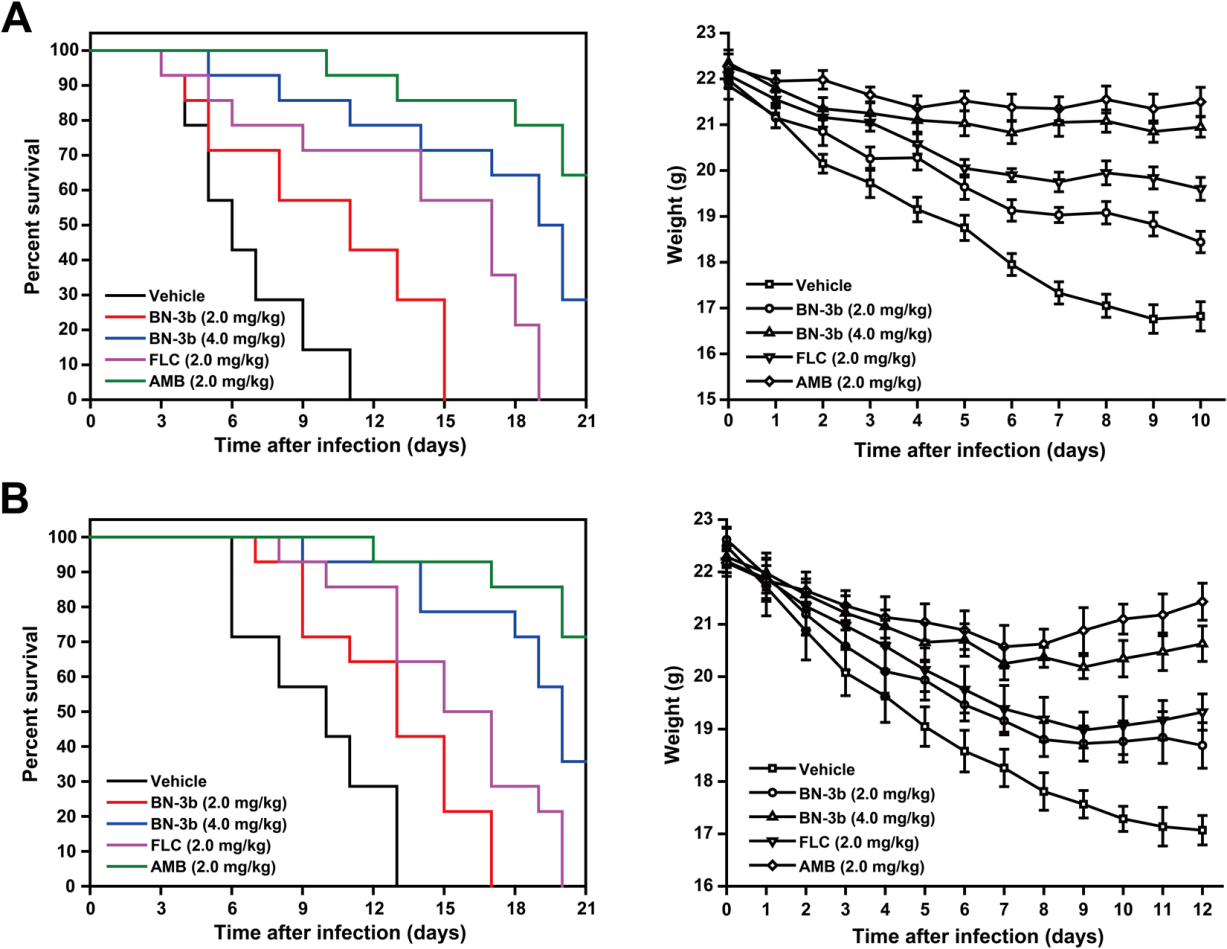
Survival and body weight of immunocompromised mice infected intravenously with *C. albicans* SC5314 (**A**) and CGMCC 2.2086 (**B**). Animals were immunocompromised by intraperitoneal injection of cyclophosphamide (100.0 mg/kg) at 3 days before and 1 day after infection. Fungal suspension (*C. albicans*: 2 × 10^5^ CFU/mouse) was inoculated into the lateral tail vein of mice, 3 days (72 h) after the intraperitoneal injection of cyclophosphamide. Animals were treated for 5 days with BN-3b (2.0 mg/kg or 4.0 mg/kg), FLC (2.0 mg/kg), AMB (2.0 mg/kg), or vehicle, starting at 24 h after infection. There were 14 animals in each treatment group.

**Figure 4:**
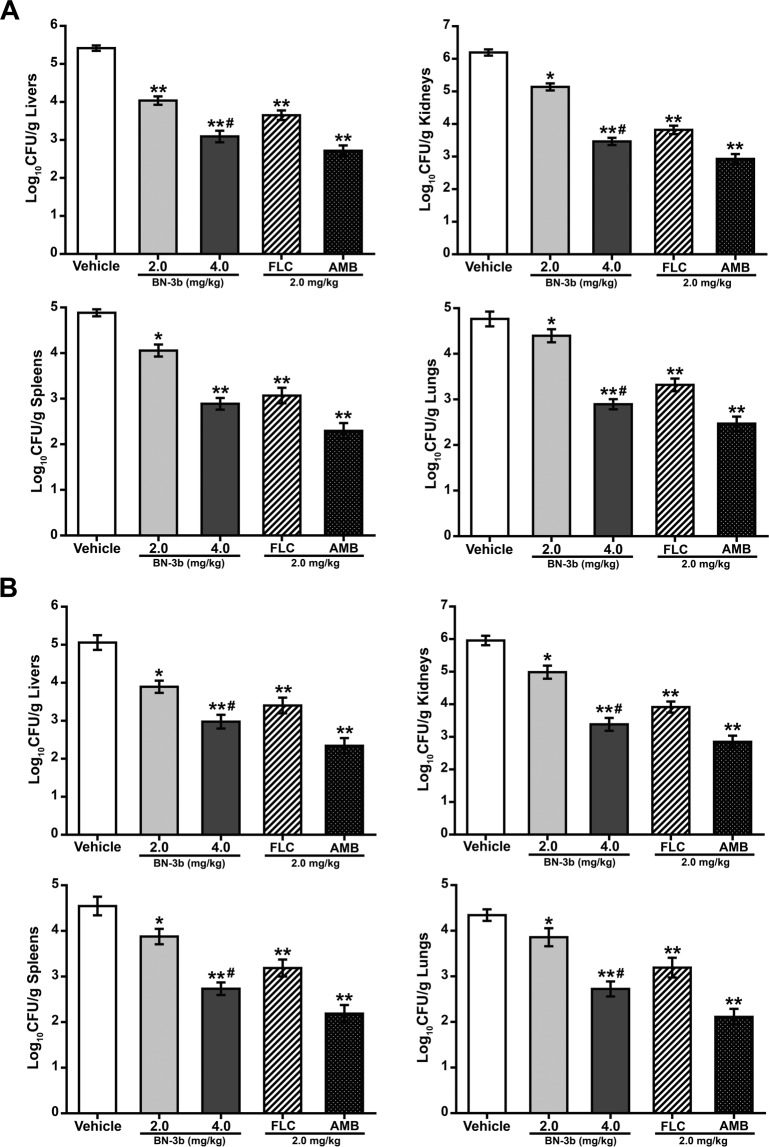
Fungal burden in liver tissues, kidney tissues, spleen tissues and lung tissues of immunocompromised mice infected intravenously with *C. albicans* SC5314 (**A**) and CGMCC 2.2086 (**B**). Intraperitoneal BN-3b (2.0 mg/kg or 4.0 mg/kg), FLC (2.0 mg/kg) and AMB (2.0 mg/kg) daily treatment was started 24 h after the *Candida* infection and lasted 5 days. Data represent the mean ± SD of 4 mice. *P < 0.05, **P < 0.01 *vs*. vehicle group; ^#^*p* < 0.05 *vs*. FLC group. CFU, Colony-forming units.

To evaluate the antifungal effect of BN-3b against *C. albicans in vivo*, we used two wild-type strains of *C. albicans* (*C. albicans* SC5314 and CGMCC 2.2086) in a model of disseminated candidiasis in mice. Percent survival over time for BN-3b, AMB, and FLC are shown in Fig. 3. All animals in the vehicle treatment group died by day 11 after infection with *C. albicans* SC5314, or day 13 with strain CGMCC 2.2086. Survival times for all groups of treated mice were significantly prolonged compared to the vehicle group. BN-3b or FLC treatments at dose of 2.0 mg/kg survived through days 15 and 19 for *C. albicans* SC5314-infected mice, respectively; days 17 and 20 for strain CGMCC 2.2086, respectively. The percent survival at day 21 after challenge of mice with 4.0 mg/kg BN-3b or 2.0 mg/kg AMB treatment were 28.6% and 64.3% for strain SC5314, respectively; 35.7% and 71.4% for strain CGMCC 2.2086, respectively (Fig. 3). In order to evaluate the impact of drugs on the body weight of mice, we also monitored the body weight of mice treated with BN-3b, AMB, FLC, or vehicle. The mean weight of mice prior to *C. albicans* SC5314 and CGMCC 2.2086 inoculations were 22.10 ± 0.30 g and 22.36 ± 0.31 g, respectively. After inoculation with *C. albicans*, a dramatic decrease in the body weights of vehicle-treated mice (Fig. 3). In contrast, body weights for all groups of treated mice were significantly higher compared to the vehicle treatment groups (*P* < 0.05). Figure 4 presents the fungal burdens in livers, kidneys, spleens, and lungs of mice treated with vehicle, BN-3b (2.0 mg/kg or 4.0 mg/kg), AMB (2.0 mg/kg), and FLC (2.0 mg/kg) by intraperitoneal injection. The BN-3b significantly reduced the number of CFU/g of liver tissues, kidney tissues, spleen tissues, and lung tissues in mice infected by *C. albicans* compared with vehicle treatment, and it was in a dose-dependent manner (Fig. 4). The results indicated that BN-3b significantly prolonged survival and decreased the fungal burdens of livers, kidneys, spleens and lungs in mouse model of disseminated candidiasis.

### BN-3b enhanced the ROS production

**Figure 5:**
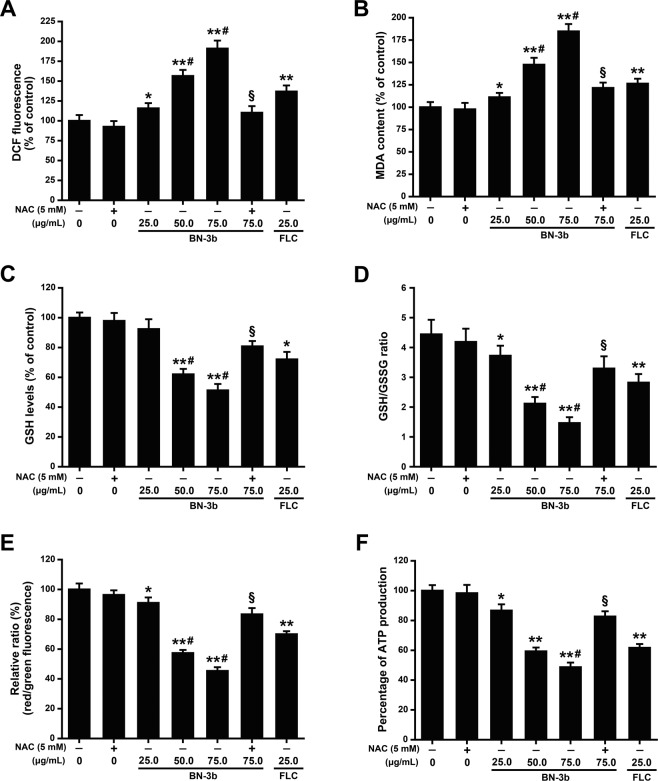
Oxidative damage induced by endogenous ROS involved in the antifungal activity of BN-3b. *C. albicans* SC5314 cells were treated with 0, 25.0, 50.0, 75.0 μg/mL BN-3b, 5 mM NAC, 5 mM NAC + 75.0 μg/mL BN-3b, respectively. (**A**) BN-3b induced ROS generation. (**B**) BN-3b increased lipid peroxides MDA. (**C**) The content of GSH. (**D**) The ratio of GSH/GSSG. (**E**) The intracellular MMP levels. The MMP was measured using a JC-1 fluorescent probe, and the JC-1 red/green fluorescence intensity ratio was used to represent MMP. (**F**) The intracellular ATP content. The results were represented as the mean ± SD from three independent experiments. *P < 0.05, **P < 0.01 *vs*. control group; ^#^*p* < 0.05 *vs*. FLC group; ^§^*p* < 0.01 *vs*. 75.0 μg/mL BN-3b group.

Intracellular ROS production was detected by using the oxidant-sensitive probe 2′,7′-dichlorofluorescin diacetate (DCFH-DA)^9^. Generation of ROS was monitored by incubation of BN-3b at 25.0, 50.0, and 75.0 μg/mL with the *C. albicans* SC5314 cells for 8 h, respectively. As shown in Fig. 5A, the ROS production induced by BN-3b increased significantly in a dose-dependent manner (*p* < 0.01), which was in turn attenuated by the addition of antioxidant *N*-acetylcysteine (NAC). The data indicated that BN-3b promoted ROS production in *C. albicans*.

### Phospholipid peroxidation of *C. albicans* induced by BN-3b

Overproduction of ROS lead to phospholipid peroxidation of membrane^9^. MDA was one of the final products of phospholipids peroxidation and could directly reflect the level of cell membrane damage^19^. To further determine the involvement of oxidative damage induced by ROS in BN-3b antifungal activity, we examined phospholipid peroxidation levels of *C. albicans* SC5314 cells treated with or without BN-3b by measuring the content of MDA. As shown in Fig. 5B, the content of MDA were significantly increased in a dose-dependent manner after treated with BN-3b compared with the control (*p* < 0.01), which was reduced by the addition of NAC. These findings indicated that BN-3b could stimulate ROS accumulation and thus resulted in *C. albicans* membrane phospholipid peroxidation.

### Effect of BN-3b on GSH

GSH plays a vital role in the protection of yeast cells against damage induced by oxidative stress^20^. As overproduction of ROS may consume GSH, we therefore examined the GSH levels of *C. albicans* SC5314 cells after treated with BN-3b. As shown in Fig. 5C, the content of GSH were significantly reduced in a dose-dependent manner after treated with BN-3b compared with the control (*p* < 0.01). Moreover, we also found that the ratio of GSH/GSSG were significantly decreased in the BN-3b treatment groups (Fig. 5D). These results further confirmed that oxidative damage induced by ROS was involved in the antifungal activity of BN-3b.

### Effect of BN-3b on MMP and ATP synthesis

In general, excessive ROS production triggered the mitochondria dysfunction^21^. To investigate whether BN-3b affected the function of mitochondria, we determined the intracellular MMP level and ATP production of *C. albicans* SC5314 treated with or without BN-3b. The MMP was measured using a JC-1 fluorescent probe, and the ratio of red/green fluorescence intensity represents MMP^22^. Our results showed that the level of MMP was significantly decreased in a dose-dependent in the BN-3b treated groups (Fig. 5E). The JC-1 red/green fluorescence intensity ratio decreased to 91.0 ± 3.6% (*p* < 0.05), 57.3 ± 2.1% (*p* < 0.01), and 45.3 ± 2.5% (*p* < 0.01), when the *C. albicans* SC5314 cells were treated with 25.0, 50.0, and 75.0 μg/mL BN-3b, respectively.

In addition, ATP content is one of the important indexes of mitochondrial activity^21^. As shown in Fig. 5F, the content of intracellular ATP decreased significantly in a dose-dependent manner after treatment with BN-3b. The content of intracellular ATP decreased to 86.7 ± 4.2% (*p* < 0.05), 59.3 ± 2.5% (*p* < 0.01), and 48.7 ± 3.1% (*p* < 0.01), respectively, when the *C. albicans* SC5314 cells were exposed to 25.0, 50.0, and 75.0 μg/mL BN-3b. At the same time, the decrease of intracellular ATP content caused by BN-3b could be in turn attenuated by the addition of antioxidant NAC. The above data suggested that the mitochondria function was impaired in BN-3b treated cells.

### Effect of BN-3b on the hyphal formation in *C. albicans*

**Figure 6:**
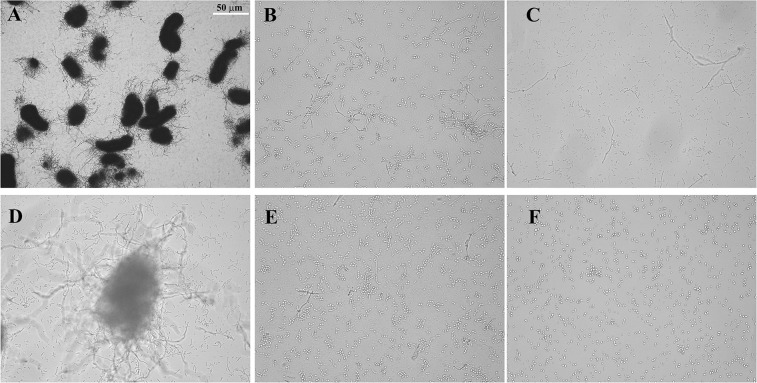
Hyphal formation of *C. albicans* SC5314 cells. *C. albicans* SC5314 cells were treated with vehicle (**A**, control), FLC (**B**, 25.0 μg/mL), AMB (**C**, 2.0 μg/mL), and BN-3b (**D**, 25.0 μg/mL; **E**, 50.0 μg/mL; **F**, 75.0 μg/mL) in YCB/FBS medium. Hyphal formation of *C. albicans* cells was obviously inhibited by BN-3b. The white bar represents a length of 50 μm.

The ability to switch from yeast to hypha was important for virulence of *C. albicans*^23^. *C. albicans* SC5314 cells incubated with vehicle or different concentrations of BN-3b (25.0, 50.0, and 75.0 μg/mL) for 8 h, and then observed by microscopy. In the vehicle treated group, large numbers of hyphae were observed in *C. albicans*. In contrast, BN-3b markedly inhibited the hyphal formation of *C. albicans* in a dose-dependent manner (Fig. 6). Especially, BN-3b completely inhibited the hyphal formation of *C. albicans* at the concentration of 75.0 μg/mL (Fig. 6F).

### Effect of BN-3b on expression of virulence-related genes

**Figure 7:**
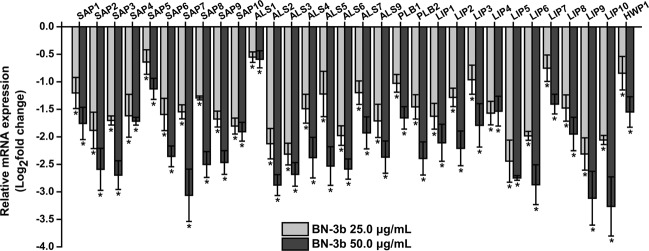
RT-qPCR analysis of virulence-related genes. RT-qPCR of *C. albicans* SC5314 treated with 25.0 μg/mL (light grey bars) or 50.0 μg/mL (dark grey bars) of BN-3b for 8 h. Cells treated with DMSO (0.1%) were used as control; *ACT1* gene was used as the internal control. Data represent the mean ± SD of three independent determinations. Significant differences from the control were indicated by *p < 0.01. The y-axis scale was log_2_ fold change.

The effect of BN-3b on the virulence-related genes (Supplementary Table S1) was examined in *C. albicans* SC5314 using RT-qPCR. RT-qPCR analyses revealed that the expressions of virulence factors of *C. albicans* SC5314 were significantly down-regulated in a dose-dependent manner after treated with BN-3b compared with the control (*p* < 0.01) (Fig. 7). These data indicated that BN-3b could exert additional anticandidal activity by inhibiting the expression of virulence factors (*SAP*s, *PLB1*, *PLB2*, *HWP1*, *ALS*s, and *LIP*s) in *C. albicans*.

## Discussion

The kill curves study indicated that BN-3b significantly reduced the cell viability of *C. albicans* in a dose-dependent manner (Fig. 1B). Following, we evaluated the *in vivo* antifungal activity of BN-3b by establishing the systemic mouse model of disseminated candidiasis. The results revealed that BN-3b significantly prolonged survival and decreased fungal burdens in mouse model of disseminated candidiasis. Based on the good antifungal activity of BN-3b *in vivo* and *in vitro*, we further discussed its action mechanism.

Increasing evidence demonstrated that oxidative damage induced by endogenous ROS was involved in the antifungal activity of antifungal agents^7–12,24^. Kobayashi *et al*. reported that the ROS production of *C. albicans* were significantly increased in a dose-dependent manner after treated with 0.125 (MIC), 1.25, and 12.5 μg/mL miconazole, respectively. FLC treatment also enhanced ROS production, especially in the 5 and 50 μg/mL groups^25^. In another study, Li *et al*. showed that treatment with 8 μg/mL of AMB significantly increased the ROS production in *C. albicans*^26^. According to the literatures and our experiment results, we used 25 μg/mL FLC or (and) 2 μg/mL AMB as positive controls for the antifungal mechanism research in the current study.

Excessive ROS may lead to oxidative damage of nucleic acids, proteins, and lipids, and ultimately lead to cell death^19^. In this study, we found that BN-3b stimulated ROS production in *C. albicans* cells (Fig. 5A). To further confirm the ROS production, we examined the intracellular MDA production and GSH concentration. The excess ROS produced will react with cell membranes, produce lipid peroxide radical, and eventually form MDA^27^. Our current study showed that the production of MDA were significantly increased in a dose-dependent manner in the BN-3b treated cells (Fig. 5B). GSH protects the cells from oxidative damage by scavenging ROS^27^. Thus, the generation of excess ROS may consume GSH. In fact, our results indicated that the content of GSH were significantly reduced in a dose-dependent manner in the BN-3b treated cells (Fig. 5C). Besides, BN-3b treatment could markedly decrease the ratio of GSH/GSSG (Fig. 5D). Conversely, antioxidant NAC could significantly attenuate BN-3b induced oxidative stress. These results indicated that BN-3b treatment impaired the balance of antioxidant system in *C. albicans* cells. In general, the overproduction of ROS might damage the membranes. Consistent with this, the cell membrane of *C. albicans* appeared obvious shrinkage and breakage (Fig. 2D–F) after exposure to BN-3b. Collectively, these data strongly suggested that BN-3b induced the endogenous ROS-mediated oxidative damage and destroyed the cell membrane, ultimately resulted in cell death.

The above results confirmed that BN-3b stimulated ROS accumulation in *C. albicans*. The excessive ROS production can lead to mitochondrial dysfunction, mainly characterized by the loss of MMP and decrease of ATP generation^28^. Our current study showed that the MMP levels of *C. albicans* cells were significantly reduced in a dose-dependent manner after treated with BN-3b. Furthermore, the results also showed that the intracellular ATP content was significantly decreased after treated with BN-3b compared with the control. These results indicated that the mitochondria function was impaired in BN-3b treated cells.

BN-3b belongs to the BN ester analogue and was synthesized by our research group. In comparison with BN, the cytotoxicity of BN-3b was significantly decreased. More significantly, BN-3b displayed potent antifungal activity against *C. albicans*, while BN was inactive^5^. It is generally accepted that the multiple bioactivities of BN were associated with the inhibition of threonyl-tRNA synthetase (ThrRS)^5,6^. The molecular docking study indicated that the side chain of BN-3b was projected deeper into the bottom of binding pocket in ThrRS than BN, which indicated the action mechanisms of BN-3b was different from BN^5^. In the present study, we found that oxidative damage induced by endogenous ROS plays an important role in the antifungal activity of BN-3b. Besides, we speculated that the unique interaction of BN-3b with ThrRS might also play a vital role in its antifungal activity, but it needs to be further verified.

Many virulence factors of *C. albicans* are involved in the infective process, such as extracellular hydrolases production, adhesion to host tissue, and hyphal formation^13,14^. In this study, we also examined the mRNA level of the virulence-related genes after exposure to BN-3b using RT-qPCR in *C. albicans*. The three most significant extracellular hydrolases produced by *C. albicans* are the SAPs, LIPs, and phospholipase B (PLBs)^29^. *C. albicans* SAPs, encoded by a multigene family^15^ (*SAP1* to *SAP10*), contribute to pathogenesis by digestion of host cell membranes and molecules of the host immune system^16^. *C. albicans* LIPs, encoded by at least ten members (*LIP1* to *LIP10*), contribute to the provision of nutrients and promote fungal penetration of host barriers^17^. Phospholipase B encoded by at least two genes (*PLB1* and *PLB2*) also contributes to the pathogenicity of *C. albicans* by abetting the fungus in damaging and traversing host cell membranes^15,29^. The results of our study showed that all of these genes were significantly down-regulated after BN-3b treatment (*p* < 0.01). The pathogenic potential of *C. albicans* was positively correlated with its adhesive capacity of the organism^30^. Currently, most studies focus on two well-characterized adhesins, Hwp1 and the ALS family^29^. The *C. albicans ALS* family has eight members (*ALS1* to *ALS7*, and *ALS9*), each encodes a large glycoprotein whose function is adhesion to host^31^. Furthermore, it has been suggested that some ALS proteins were involved in growth-related functions^17^. Our current study showed that these genes were significantly down-regulated after BN-3b treatment (*p* < 0.01). Therefore, down-regulation of these genes not only affect the infection ability of *C. albicans* but also affect the cell proliferation of *C. albicans*. *HWP1*, a hypha-specific gene, which encodes a cell-surface adhesin that promotes interactions between *C. albicans* and host cells^17,29^. It is interesting to note that the mRNA level of *HWP1* decreased significantly after treatment with BN-3b. Hyphae formation plays a key role in *C. albicans* pathogenicity^13,32^. In this study, we found that BN-3b strongly inhibited the hyphal formation of *C. albicans*. Thus we suggested that BN-3b could inhibit the yeast-to-hypha transition and down-regulate the expressions of virulence factors to weaken the pathogenicity of *C. albicans*.

Our results indicated that BN-3b exerts antifungal effect through increasing the generation of ROS, decreasing the MMP, reducing the intracellular ATP level, and destroying the cell membrane. In addition, BN-3b exerts added anticandidal activity by inhibiting the yeast-to-hypha transition and down-regulating the expressions of virulence factors. These findings suggested that BN-3b may be a promising lead for the development of antifungal agent.

## Materials and Methods

### Drugs

BN-3b was synthesized according to our previous report^5^. BN-3b, AMB (Sigma-Aldrich) and FLC (Solarbio Science & Technology Co., Ltd., Beijing, China) were dissolved in dimethyl sulphoxide (DMSO) to prepare 150.0 mg/mL, 10.0 mg/mL and 10.0 mg/mL of stock solutions and stored at −20 °C. Working solutions of BN-3b, AMB, and FLC were prepared by diluting the stock solution with DMSO prior to use^28^. *N*-acetylcysteine (Beyotime Biotechnology Co., Shanghai, China) was dissolved in phosphate-buffered saline (PBS) to prepare 0.5 M of stock solution and stored at −20 °C. Cyclophosphamide (CY, J&K Scientific Ltd., Beijing, China) was dissolved in 0.9% NaCl sterile solution to prepare 50.0 mg/mL of stock solution and stored in the dark at 4 °C.

### Organism and culture conditions

*C. albicans* SC5314 (ATCC MYA-2876), *C. albicans* CGMCC 2.2086, *C. parapsilosis* (ATCC 22019), *Cryptococcus neoformans* (ATCC 208821), and *Rhizoctonia solani* (CGMCC 3.7376) were obtained from China General Microbiological Culture Collection Center. *Aspergillus niger* (CCTCC AF 93021), *A. fumigatus* (CCTCC AF 93048), *A. alternata* (CCTCC AF 93103), *F. oxysporum* (CCTCC AF 93247), and *B. cinerea* (CCTCC AF 93110) were obtained from China Center for Type Culture Collection. All strains were maintained on YPD or YPDA^33^. Hyphal development was induced using the yeast carbon base/fetal bovine serum (YCB/FBS) liquid media, containing 1.17% (w/v) yeast carbon base (BD Biosciences), 1% (w/v) glucose, and 10% (v/v) fetal bovine serum (Gibco BRL, USA).

### *In vitro* assay for antifungal activities

The MICs of BN-3b were determined using the method described by CLSI guidelines^34^. FLC and AMB were used as positive controls. The tested compounds were dissolved in DMSO and 2-fold serially diluted to eight different concentrations^5^ (10-0.078 mg/mL for BN-3b, 0.8–0.00625 mg/mL for FLC and AMB). The above samples (1 μL) and 100 μL of prepared fungal suspensions (in RPMI-1640 medium) containing 2 × 10^3^ cfu/mL of fungus were added to each well of 96-well microtiter plates^5^. The vehicle treated wells were used as control. The plates were incubated for 48 h at 28 °C, and the absorbance was recorded spectrophotometrically at 620 nm using a microplate reader (BioTek Synergy H1, BioTek Instruments, Inc., Vermont, USA). The MICs of the BN-3b and AMB were defined as the lowest concentrations that completely inhibited visual growth of an organism^35^. The concentration of FLC which caused a 80% reduction in the absorbance compared to the control was considered as the MIC^36^.

### The viability assay of *C. albicans*

Fungal suspensions at 6 × 10^5^ CFU/mL in YPD liquid medium were exposed to BN-3b (0, 0.5 × MIC, MIC, 1.5 × MIC, 2 × MIC, and 3 × MIC), and incubated for 2, 4, 6, 8, 10 or 12 h at 37 °C. Then, yeasts were washed twice with PBS, diluted serially (1:10) and spread on sabouraud dextrose agar (SDA) plates. After 48 h of incubation at 37 °C, the viability of *C. albicans* was determined by colony counting^12^. Each concentration was performed with three biological replicates.

### Ultrastructure analysis

*C. albicans* SC5314 cells treated with BN-3b (0, 25.0, 50.0, or 75.0 μg/mL) for 8 h. The samples were treated according to our previous report^33^. The ultrastructure of *C. albicans* was observed under a Hitachi H-7650 transmission electron microscope (Tokyo, Japan). The treatment of AMB (2.0 μg/mL) and FLC (25.0 μg/mL) served as positive controls.

### *In vivo* assay for antifungal activity

The male BALB/c mice (weight, 20–25 g) were purchased from Changsheng biotechnology Co., Ltd. (Liaoning, China). All procedures were conducted in accordance with the Guide for the Care and Use of Laboratory Animals (Ministry of Science and Technology of China, 2006)^6^ and approved by the Laboratory Ethics Committees of College of Life and Health Sciences of Northeastern University^37^.

In order to quickly establish mouse model of disseminated candidiasis, all mice used in this study were received CY at 100 mg/kg body weight administered intraperitoneally 3 days before and 1 day after infection^38^. Fungal suspension (*C. albicans*: 2 × 10^5^ CFU/mouse in volume of 0.1 mL) was inoculated into the lateral tail vein of mice, 3 days (72 h) after the intraperitoneal injection of CY. Mice were randomly separated into five groups (n = 14 per group). BN-3b therapy with a dose of 2.0 mg/kg or 4.0 mg/kg of body weight daily, AMB or FLC therapy with a dose of 2.0 mg/kg of body weight daily by intraperitoneal injection were initiated at 24 h after infection, and continuous administration for 5 days. Control group were treated with vehicle (60% 1,2-propanediol) in the same way. Twenty-four hours after the last dose of antifungal agent, four mice of each group were sacrificed to determine the fungal burden in the liver, kidney, spleen, and lung. The organs were excised by a sterile technique, weighed, and homogenized in 5 mL of sterile saline^9^. The homogenates were serially 10-fold diluted in sterile saline, and 100 μL was plated on SDA^39^. Plates were incubated for 48 h at 35 °C and the number of CFU/g of tissue was calculated^9^. In the study of the survival rate and body weight in mice, fourteen mice in each group were monitored daily until 15 days after the end of therapy (21 days after infection).

### Measurement of ROS production

Followed the methods as previously described, *C. albicans* SC5314 (10^7^ CFU/mL) incubated with 40 μM DCFH-DA at 37 °C for 60 min in the dark^9^, the cells were collected, washed twice and then diluted to 6 × 10^5^ CFU/mL with YPD^12^. After that, a series of BN-3b were added and incubated at 37 °C for 8 h. And then washed and re-suspended in 100 μL of PBS. The fluorescence intensity of a cell suspension (100 μL) containing 10^7^ cells was measured using a microplate reader with excitation at 480 nm and emission at 530 nm^9^. The NAC (5 mM) treated cells were used as negative control, and FLC (25.0 μg/mL) as positive control.

### Measurement of phospholipid peroxidation

We examined phospholipid peroxidation of membranes by measuring the levels of MDA^9^. Briefly, *C. albicans* SC5314 (6 × 10^5^ CFU/mL) incubated with different concentrations of BN-3b for 8 h. And cells (3 × 10^7^) were collected and washed three times with PBS. The pellet was resuspended in 200 μL PBS and freezed overnight at −80 °C, then samples were heated in a boiling bath for 5 min and, cooled to room temperature and added the reagents of MDA Assay Kit (Jiancheng Institute of Biotechnology, Nanjing, China). Finally, the absorbance of the supernatant was measured at 532 nm using a microplate reader^27^. The FLC (25.0 μg/mL) treated cells were used as positive control.

### GSH/GSSG assay

*C. albicans* SC5314 (6 × 10^5^ CFU/mL) incubated with different concentrations of BN-3b (0, 25.0, 50.0, and 75.0 µg/mL) for 8 h. And cells (3 × 10^7^) were collected and washed three times with PBS. The pellet was resuspended in 200 μL PBS and freezed overnight at −80 °C, then samples were heated in a boiling bath for 5 min and, cooled to room temperature, then the supernatants were collected by centrifugation. GSH and GSSG levels were quantified using GSH/GSSG Assay Kit (Jiancheng Institute of Biotechnology, Nanjing, China) according to the manufacturer’s protocol. The absorbance was measured at 405 nm. The FLC (25.0 μg/mL) treated cells were used as positive control.

### Measurement of MMP

The MMP was measured with a Mitochondrial Membrane Potential Assay Kit with JC-1 (Beyotime Biotechnology Co., Shanghai, China)^22^. *C. albicans* SC5314 (6 × 10^5^ CFU/mL) incubated with vehicle or different concentrations of BN-3b (25.0, 50.0, and 75.0 µg/mL) for 8 h. Then cells (3 × 10^7^) were incubated with a JC-1 staining solution at 37 °C for 20 min in the dark, washed twice with PBS, and resuspended in the buffer^28^. Green (excitation/emission wavelength: 514/529 nm) and red (excitation/emission wavelength: 585/590 nm) fluorescence were detected on a microplate reader^28^. The ratio of red/green fluorescence intensity represents MMP^22^.

### Synthesis of ATP assay

The cellular ATP level was detected using an ATP Bioluminescence Assay Kit (Beyotime Biotechnology Co., Shanghai, China)^28^. *C. albicans* SC5314 (6 × 10^5^ CFU/mL) incubated with vehicle or different concentrations of BN-3b (25.0, 50.0, and 75.0 µg/mL) for 8 h. After that, cells (5 × 10^6^) from each culture were lysed and centrifuged, and then 100 μL of ATP detection working solution as well as 50 μL supernatant were added to 96-well plate, and then luminescence was measured on a microplate reader^28^.

### Hyphal formation assay

The hyphal formation of *C. albicans* induced by YCB/FBS medium^40,41^. *C. albicans* SC5314 (6 × 10^5^ CFU/mL) incubated with different concentrations of BN-3b for 8 h at 37 °C. The hyphal formation of *C. albicans* was recorded with a microscope with the magnification of 400×. The treatment of AMB (2.0 μg/mL) and FLC (25.0 μg/mL) severed as positive controls.

### RNA extraction and RT-qPCR

*C. albicans* SC5314 cells were treated with vehicle and BN-3b (25.0 or 50.0 μg/mL) in YCB/FBS medium as the same method above. Total RNA was extracted with AxyPrep Multisource Total RNA Miniprep Kit (Axygen, China) and reverse transcribed with GoScript^TM^ reverse transcription system (Promega, USA) by following the manufacturer’s instructions. RT-qPCR was conducted according to our previous report^33^. RT-qPCR was performed with the primer sets listed in Supplementary Table S1. *ACT1* gene was used as the internal control. Fold changes were calculated using the 2^−△△Ct^ method^33^.

### Statistical analysis

All data were represented as the mean ± standard deviation (SD) from at least three independent experiments. Statistical analysis was determined by using one-way analysis of variance (ANOVA) followed by Tukey’s multiple comparison test, *p* ≤ 0.05. The SPSS 17.0 statistical software package was used for data analysis.

## Supplementary information

Supplementary Information.

## Data Availability

The datasets that were generated and/or analysed during the current study are freely available from the corresponding author on a request.
