# Supplementary material for: A semisynthetic borrelidin analogue BN-3b exerts potent antifungal activity against Candida albicans through ROS-mediated oxidative damage
Source: Sci Rep. 2020 Mar 19;10:5081. doi: 10.1038/s41598-020-61681-0 (PMC7081223; doi:10.1038/s41598-020-61681-0)
Supplement: Supplementary file 1 — Supplementary Information. [file 41598_2020_61681_MOESM1_ESM.pdf]

Supplementary Information

A semisynthetic borrelidin analogue BN-3b exerts potent antifungal activity against *Candida albicans* through ROS-mediated oxidative damage

Hao Su, Caijuan Hu, Bixuan Cao, Xiaodan Qu, Peipei Guan, Yu Mu, Li Han* & Xueshi Huang*

Institute of Microbial Pharmaceuticals, College of Life and Health Sciences, Northeastern University, Shenyang 110819, P. R. China

Tel: 0086-24-83656106. E-mail: huangxs@mail.neu.edu.cn (X. Huang).

Tel: 0086-24-83656122. E-mail: hanli@mail.neu.edu.cn (L. Han).

Supplementary Information Content:

Primers used in this study.

Table S1 Primers used in this study

Genes	Description	GenBank accession no.	Primer	Sequence(5′to 3′)
SAP1	secretory aspartyl proteinase SAP1p	XM712894	Forward	GGTTCATTGATTGCTGTTCC
			Reverse	CCATCCAGTTTCAATTCAGC
SAP2	secretory aspartyl proteinase SAP2p	XM705955	Forward	CCAAGTGGTTCATCAGCTTCA
			Reverse	AACACCACCAAATCCAACGG
SAP3	secretory aspartyl proteinase SAP3p	XM718117	Forward	AGTTTCATGTCAAGCTGGTCAA
			Reverse	TGTCCCTTGTGAAGTAGTTCCA
SAP4	secretory aspartyl proteinase SAP4p	XM712895	Forward	GCGTTATTGTTGACACTGGC
			Reverse	GCAGCTGGAGAATAGGAACC
SAP5	secretory aspartyl proteinase SAP5p	XM714172	Forward	CCCGCACTTCCCAAAATTTG
			Reverse	AACACCACCAATACCAACGG
SAP6	secretory aspartyl proteinase SAP6p	XM714129	Forward	ACGAAGCTACCAGAACTCCT
			Reverse	GGCCTTGTCAATACCACCAA
SAP7	secretory aspartyl proteinase SAP7p	XM708473	Forward	AGTGGTGCTGAATTGTGTGT
			Reverse	GATAGGAACAACGGCATGGT
SAP8	secretory aspartyl proteinase SAP8p	XM714848	Forward	CAGCAGCAACAACAACAACA
			Reverse	CAGCAGAATCAACCACCCAT
SAP9	secretory aspartyl proteinase SAP9p	XM707599	Forward	TCGAATTCAGTTGGCGCTTA
			Reverse	ACATGTACGACGAGCTTGAC
SAP10	secretory aspartyl proteinase SAP10p	XM712150	Forward	CCCAACCCAGTTCAACCATT
			Reverse	TGCACTCAATATGGCGATCC
ALS1	agglutinin-like ALS1 protein	XM712917	Forward	TGTTACTGGTGGAGCTGTTG
			Reverse	TGTGTTGGTTGAAGGTGAGG
ALS2	agglutinin-like ALS2 protein	XM706992	Forward	TCCTATGCTACGACCACCAC
			Reverse	TGACAGTTGGGTTTGGAGGT
ALS3	agglutinin-like ALS3 protein	XM707573	Forward	AGCCACCAAATTACACGGTT
			Reverse	GTGGGGCTGTGATAGTTGAG
ALS4	agglutinin-like ALS4 protein	XM705333	Forward	AACTACCGTGACTGCTCCTC
			Reverse	TGATAACCGTGTCAGTGCCT
ALS5	agglutinin-like ALS5 protein	XM712914	Forward	ATTTGACTGCCGATGGTGTT
			Reverse	GTAACCGTACCCAAAGCCTT
ALS6	agglutinin-like ALS6 protein	XM710986	Forward	CAAACTCACAGCCAACCACA
			Reverse	GGAAGGATGTTTAGTGGCGG
ALS7	agglutinin-like ALS7 protein	XM710972	Forward	GAGTTTGCCTGTGTGAATGG
			Reverse	TTTGGTTTTCTGGAGTCGGG
ALS9	agglutinin-like ALS9 protein	XM712918	Forward	GCAGGAGACACGTTTACCTT
			Reverse	TGACCGTACCAGAAGCCTTA
PLB1	phospholipase B1	XM708770	Forward	GCGGAGGGTATAGAGCAATG
			Reverse	ACTAACCACGATCCACCTGA
PLB2	phospholipase B2	XM708772	Forward	ATCCAATACTAGCCGCGTTG
			Reverse	GGATCCCATGAACCCACTTC
LIP1	*C. albicans* secretory lipase 1	XM718224	Forward	GCTGCTACTGACAATTTGCG
			Reverse	GGGAAAGCTCTATCATCGCC
LIP2	*C. albicans* secretory lipase 2	XM718207	Forward	TGCTGTGGGAAGACAATCAG
			Reverse	CCAGTAGCCAAAGATCCACC
LIP3	*C. albicans* secretory lipase 3	XM718258	Forward	AATTGGTGTGATTGGGGCAT
			Reverse	AACCATGTTATAGCTGCGGG
LIP4	*C. albicans* secretory lipase 4	XM707315	Forward	ATCACTGGTGTTTTCGTCCC
			Reverse	ATGAAGCGAGTTTGGAAGGG
LIP5	*C. albicans* secretory lipase 5	XM711841	Forward	ATGCTGCCGTTGGTGATATT
			Reverse	TCTTGGATGGATCAGCGTTG
LIP6	*C. albicans* secretory lipase 6	XM718226	Forward	GGCGTAGGAAGACAATCAGG
			Reverse	GGCAAACGATCCTCCAGAAT
LIP7	*C. albicans* secretory lipase 7	XM711361	Forward	CAGAGCTTGAATTGGTGGGA
			Reverse	AATCTGTCCCTGAAATGGCG
LIP8	*C. albicans* secretory lipase 8	XM706575	Forward	TGTGCTCCTTCCTATGCTCT
			Reverse	CAACACAGCTTGACCAGACT
LIP9	*C. albicans* secretory lipase 9	XM711834	Forward	CACCCAGGCTGAAACGTATT
			Reverse	ACTTCAAACTTGCGCGAATG
LIP10	*C. albicans* secretory lipase 10	XM718225	Forward	GACTGCTCACCTTCATACGG
			Reverse	TTGCCTTCCCAGACTGTCTA
HWP1	hyphal-specific cell wall protein aka ECE2	XM707905	Forward	CCTGCTCCTGAAATGACTCC
			Reverse	GGAGAAGAAGAAGCACCTGG
ACT1	Actin	XM717232	Forward	TGGAAGCTGCTGGTATTGAC
			Reverse	TCCTTTTGCATACGTTCAGC
